# Apolipoprotein A5 gene polymorphism (rs662799) and cardiovascular disease in end-stage kidney disease patients

**DOI:** 10.1186/s12882-022-02925-1

**Published:** 2022-09-07

**Authors:** Jerry Jacob, Sylwia Boczkowska, Wojciech Zaluska, Monika Buraczynska

**Affiliations:** 1 Hope Medical Institute, Newport News, VA USA; 2 Department of Nephrology, Medical University of Lublin, Jaczewskiego 8, 20-950 Lublin, Poland

**Keywords:** Apolipoprotein A5, End-stage kidney disease, rs662799 polymorphism, Genotyping, Risk allele, Triglycerides

## Abstract

**Background:**

Plasma triglyceride (TG) levels are a significant risk factor for cardiovascular disease (CVD). The *APOA5* gene is one of the crucial factors in plasma TG metabolism regulation. The rs662799 polymorphism in the *APOA5* gene has been reported to be associated with cardiovascular disease. The goal of this study was to evaluate the potential association of this variant with CVD in patients with end-stage kidney disease.

**Methods:**

In this case–control study the polymorphism was analyzed using the PCR–RFLP method in 800 consecutive patients with ESKD and 500 healthy controls. The genotype and allele distribution was compared between subgroups of patients with CVD (552) versus those without CVD (248).

**Results:**

The frequency of the minor allele (C) in the healthy individuals was 9% compared to 12% in ESRD group (*p* = 0.09). The difference between groups was slightly higher for CC homozygote (3.5% versus 1.6%, *p* = 0.042). The ESKD patient group was analyzed according to the presence or absence of CVD. The significant differences in the polymorphism distribution were revealed in this analysis. The frequency of the C allele in the CVD + subgroup was 14% compared to 6% in CVD- patients (*p* = 0.001). In the CVD + subgroup the ORs (95% CI) for the C allele and CC genotype were 2.41 (1.61–3.6), *p* < 0.001 and 3.13 (1.07–9.14), *p* = 0.036, respectively. This indicates to the association of the variant C allele with cardiovascular disease in ESKD patients. The CC homozygotes have a threefold higher odds of CVD compared to TT homozygotes. The highest frequency of the C allele (18%) was observed in subgroup of patients with diabetic nephropathy, with OR (95% CI) 3.40 (2.13–5.43), *p* < 0.001.The presence of minor allele (CC and CT genotypes) was significantly associated with increased plasma triglyceride levels (*p* < 0.001 for both CVD + and CVD- groups).

**Conclusion:**

The present study demonstrated the effect of rs662799 polymorphism on plasma TG levels and its association with the development of cardiovascular disease in ESKD patients.

## Background

Patients with end-stage kidney disease (ESKD) are particularly susceptible to cardiovascular disease (CVD). In the United States over 45% of deaths in ESKD patients are due to CVD [1]. In comparison to general population, the incidence of cardiovascular death is 10 to 30-fold higher [2]. This increased mortality rate is only in part justified by traditional cardiovascular risk factors such as age, obesity, hypertension, hyperglycemia or hyperlipidemia [3]. Environmental and genetic factors play an equally important role in a high prevalence of CVD. With the development of high-throughput technology, the candidate gene approach and genome-wide association studies have successfully identified numerous CVD susceptibility genes, but such studies in chronic kidney disease patients are limited [4, 5].

Plasma triglycerides (TG) are considered an important risk factor for both cardiovascular [6] and renal disease [7]. Apolipoprotein A5 is involved in TG metabolism and is a significant modulator of serum TG concentration. In humans it is expressed almost exclusively in the liver [8, 9]. Overexpression of apoA5 decreases circulating TG levels, while its reduced expression causes an opposite effect [10].

Apolipoprotein A5 gene (*APOA5*) is a member of the *APOA4 / APOC3 / APOA1* apolipoprotein cluster and is located on a short arm of chromosome 11 (11q23) [11, 12]. Several single nucleotide polymorphisms (SNPs) in the *APOA5* gene have been identified. Extensively studied and important variants include -1113 T > C and S19W polymorphisms [13]. The earlier studies pointed to the associations between common variants of *APOA5* gene and differences in plasma TG levels [14–16]. These associations suggested that *APOA5* gene is a functional candidate for cardiovascular disease. Several studies have reported an association between *APOA5* gene single nucleotide polymorphisms and coronary artery disease [14, 17–20]. The *APOA5* -1113 T > C (rs662799) polymorphism, located in the promoter region of the gene, plays a major role in the development of CHD through its strong association with increased plasma TG levels [21].

Since the end-stage kidney disease patients are particularly prone to cardiovascular disease, we aimed to assess the potential association of the *APOA5* -1113 T > C polymorphism with a risk of CVD in this group of patients.

## Methods

### Patients and controls

The population enrolled in the present case–control study consisted of 800 unrelated, adult Caucasian patients with end-stage kidney disease (ESKD) (mean age 62.5 ± 14.3). Genomic DNA was obtained from individuals undergoing hemodialysis at the University Hospital and dialysis center of Medical University of Lublin between 2006 and 2019. This patient cohort was used and described in our earlier study [22]. End-stage kidney disease was defined according to KDOQI (Kidney Disease Outcomes Quality Initiative) definition, considering estimated glomerular filtration rate (eGFR) < 15 ml/min/1.73 m^2^, the presence of clinical signs of uremic syndrome, and requirement of renal replacement therapy. Patients on dialysis for < 6 months, those presented with primary or secondary immunodeficiencies, on immunosuppressive therapy, with current pregnancy, malignancy and active systemic infection were excluded from this study. This ensured well defined disease phenotype in all patients. Cardiovascular disease was diagnosed at the time of DNA sample collection in 552 patients (69%), the rest (248) were free of CVD. One or the combination of pathological phenotypes: congestive heart failure, left ventricular hypertrophy, angina pectoris, ischemic heart disease, myocardial infarction, ischemic cerebral stroke was diagnosed as cardiovascular disease. Clinical presentation of CVD was verified by relevant biochemical, radiographic, echocardiographic and vascular diagnostic criteria. Hypertension was diagnosed according to WHO criteria in 539 patients. An extended clinical assessment of all patients involved complete medical history, laboratory determinations and physical examination.

The control group consisted of 500 healthy individuals, randomly recruited among Medical University of Lublin hospital staff and blood donors who underwent health examination. This group was also used in our earlier study [22]. All presented with normal ECG and without any clinical evidence of CVD. There was no past history of kidney disease and the serum creatinine levels were tested before enrollment. Individuals with positive family history of renal or cardiovascular disease in first-degree relatives were excluded from the study. A written informed consent for participating in the present study was obtained from all subjects from patient and control groups. The protocol of the study was approved by the Ethics Committee of the Medical University of Lublin. The investigation conforms to the principles of the Declaration of Helsinki in all the procedures employed in this study.

### Determination of APOA5 rs662799 genotype

Genomic DNA was extracted from 10 ml of peripheral blood leukocytes by the standard procedure [23]. DNA concentration and quality of samples were determined in Nano Drop 2000 (Thermo Fisher Scientific USA). The rs662799 (-1131 T/C) variant in the *APOA5* gene was detected by polymerase chain reaction—restriction fragment length polymorphism (PCR–RFLP) assay, as reported earlier [24]. Slightly modified conditions for PCR reactions were applied: initial denaturation at 95 °C for 5 min was followed by 35 cycles of denaturation at 95 °C for 30 s, annealing at 55 °C for 1 min and extension at 72 °C for 1 min, with a final extension step at 72 °C for 10 min. Negative controls without DNA were used in each experiment. The PCR product of 187 bp was incubated with 5 units of Mse I restriction endonuclease (Thermo Fisher Scientific) at 37 °C for 12 h. The products of Mse I digestion were analyzed by electrophoresis in 2.5% agarose gel. The length of fragments was 167 bp + 20 bp for the T allele and 187 bp for the C allele. The case–control status of the samples was blinded during genotyping. The correctness of genotyping was validated by repeating PCR reactions for 20% of random samples. In addition, randomly selected samples (20 for each genotype) were sequenced in CEQ 8000 Genetic Analysis System (Beckman Coulter, England) to confirm the genotype reading in agarose gel. We observed 100% concordance between genotyping assays.

### Statistical analysis

The statistical data processing and analysis were conducted using SPSS version 14.0 for Windows (SPSS, Inc., Chicago, IL. USA). In the baseline characteristics, the values of normally distributed variables are represented by mean ± standard deviation (SD) or numbers / percentages where appropriate. Comparisons of discrete and continuous variables between groups were done by Student’s *t*-test and Mann–Whitney test. Potential deviation from Hardy–Weinberg balance in the distribution of genotypes was tested using a chi-square goodness-of-fit test with 1 degree of freedom. The *APOA5* rs662799 polymorphism distributions in patients with CVD and CVD-free subjects, were compared using a Pearson chi-square test of independence. For documenting the significant associations of studied polymorphism with clinical phenotype, the odds ratios (OR) with corresponding 95% confidence intervals (CI) were computed, with non-risk allele or genotype considered a reference. A multivariate logistic regression analysis was applied to investigate the genotype impact and clinical profile associated with the risk of CVD in ESKD patients and to verify the independence of observed associations. ORs were adjusted for age, gender, BMI, the presence of hypertension and diabetes mellitus. Four genetic models were considered: an additive, a co-dominant (CC or TC vs. TT), a dominant (heterozygote with homozygote for the minor allele: CC plus TC vs. TT) and recessive model (homozygote for the minor allele: CC vs. TC plus TT). Power estimations based on allele frequencies were done with the program for case–control study, available at http://pngu.mgh.harvard.edu/~purcell/gpc/. The level of statistical significance for all tests was set at *p* < 0.05.

## Results

In total, 800 patients with ESKD were genotyped with the rs662799 polymorphism in the *APOA5* gene. For the purpose of this association study they were divided into two groups, patients with CVD (*n* = 552) and those without CVD (*n* = 248). Their clinical and laboratory details are presented in Table 1. For the gender distribution, years on dialysis, total cholesterol and serum creatinine levels no significant differences were observed between CVD + and CVD- patients. Individuals with CVD were older (mean age 67.4 ± 14.2 years) than patients without CVD (mean age 58.3 ± 15.3 years). Hypertension and diabetes were more prevalent in CVD + group (both *p* < 0.001). The levels of triglycerides were higher (*p* = 0.004) and of HDL cholesterol lower (*p* = 0.002) in the CVD + group. There was also a significant difference in BMI (*p* = 0.001) between study groups. This indicates that in our study, age, diabetes, hypertension, BMI and triglyceride and HDL cholesterol are risk factors for CVD.

**Table 1:** Basic characteristics of ESKD patients according to the presence or absence of CVD

Parameters	ESKD CVD +	ESKD CVD-	*p* value
N	552	248	
Gender (M/F)	306/246	119/129	0.053
Age (years)	67.4 ± 14.2	58.3 ± 15.3	< 0.001
Years on dialysis	4.8 ± 2.9	5.1 ± 3.6	0.210
Diabetes mellitus (%)	198 (36)	55 (22)	0.001
Hypertension (%)	420 (76)	119 (48)	< 0.001
BMI (kg/m^2^)	27.2 ± 5.3	25.9 ± 5.1	0.001
SBP (mmHg)	146 ± 9	143 ± 11	< 0.001
DBP (mmHg)	83 ± 7	81 ± 12	0.003
Total cholesterol (mmol/l)	4.7 ± 2.3	4.8 ± 1.7	0.539
HDL cholesterol (mmol/l)	1.2 ± 0.4	1.3 ± 0.5	0.002
Triglycerides (mmol/l)	1.8 ± 1.3	1.6 ± 1.4	0.004
Serum creatinine (μmol/l)	761 ± 147	782 ± 164	0.072

Values are mean ± SD or numbers (%)

Variable values determined by Student’s t-test for continuous and Mann Whitney test for discrete variables

*ESKD* End-stage kidney disease, *CVD* Cardiovascular disease, *BMI* Body mass index, *SBP* Systolic blood pressure, *DBP* Diastolic blood pressure

The frequencies of the rs662799 genotypes (TT, TC and CC) among ESKD patients and healthy controls are summarized in Table 2. Both allele and genotype proportions in control group are in Hardy–Weinberg equilibrium (χ^2^ = 3.335, *p* = 0.067). The frequency of the minor allele (C) in the healthy population involved in this study was 9% compared to 12% in ESRD group (*p* = 0.09). Considering the CC homozygote, the difference between groups was statistically significant (3.5% versus 1.6%, *p* = 0.042).

**Table 2:** Distribution of rs662799 (T1131C) polymorphism in the *APOA5* gene in ESKD patients and healthy controls

	Genotypes				MAF	OR (95% CI)^a^		
	N	TT	TC	CC		C allele	CC genotype^b^	TC genotype^b^
ESKD patients	800	644 (80.5)	128 (16)	28 (3.5)	0.12	1.26 (0.97–1.64)	2.25 (1.01–4.99)	1.07 (0.78–1.45)
						*p* = 0.077	*p* = 0.045	*p* = 0.061
Controls	500	415 (83)	77 (15.4)	8 (1.6)	0.09	ref	ref	ref

Genotype distribution is shown as numbers (%). Hardy–Weinberg equilibrium: χ^2^ = 3.335, *p* = 0.067 for control group

*ESKD* End-stage kidney disease, *APOA5* Apolipoprotein A5, *MAF* Minor allele frequency

^a^Adjusted for age, sex, BMI, hypertension and diabetes

^b^Calculated versus TT genotype

The ESKD patient group was further analyzed according to the presence or absence of CVD. The distribution of the rs662799 polymorphism was compared between the subgroups of CVD + and CVD- patients. As shown in Table 3, the significant differences were observed in this analysis. A strong association of the C allele with cardiovascular disease was observed in ESKD patients. The frequency of the C allele in the CVD + subgroup was significantly higher than in CVD- subgroup (14% and 6%, respectively, with *p* = 0.001). Considering the genetic model, in the additive model (C vs. T), OR for the C allele was 2.41 (1.61–3.6), *p* < 0.001, representing 2.4-fold higher odds of CVD per copy of the C allele. In a co-dominant model (CC vs.TT), the OR for the CC genotype was 3.13 (1.07–9.14), *p* = 0.036. This represents a threefold higher odds of CVD for CC homozygotes compared to TT homozygotes. The OR for the TC genotype was 2.38 (1.47–3.85), *p* < 0.001, representing over twofold higher odds for heterozygotes. With the minor allele frequency 0.06 in CVD- subgroup and 0.14 in CVD + patients, the statistical power to detect an association of the carrier genotypes with CVD was 93.8%.

**Table 3:** Distribution of rs662799 polymorphism in the *APOA5* gene in ESKD patients with and without CVD

	Genotypes				MAF	OR (95% CI)^a^		
	N	TT	TC	CC		C allele	CC genotype^b^	TC genotype^b^
ESKD CVD +	552	423 (76.7)	105 (19)	24 (4.3)	0.14	2.41 (1.61–3.6)	3.13 (1.07–9.14)	2.38 (1.47–3.85)
						*p* < 0.001	*p* = 0.036	*p* < 0.001
ESKD CVD-	248	221 (89.1)	23 (9.3)	4 (1.6)	0.06	ref	ref	ref

Genotype distribution is shown as numbers (%)

*ESKD* End-stage kidney disease, *CVD* Cardiovascular disease, *APOA5* Apolipoprotein A5, *MAF* Minor allele frequency

^a^Adjusted for age, sex, BMI, hypertension and diabetes

^b^Calculated versus TT genotype

The rs662799 polymorphism distribution was also analyzed in ESKD subgroups with different primary kidney diseases (Table 4). The highest frequency of the C allele (18%) was observed in subgroup of patients with diabetic nephropathy, with OR (95% CI) 3.40 (2.13–5.43), *p* < 0.001.

**Table 4:** Distribution of rs662799 polymorphism in the *APOA5* gene in ESKD CVD + patients with different primary kidney disease

ESKD CVD + subgroup	Genotypes				MAF	OR (95% CI)^a^	*P* value
	N	TT	TC	CC		for C allele^b^	
CGN	157	123 (78)	28 (18)	6 (4)	0.13	2.18 (1.33–3.58)	0.002
DN	146	102 (70)	34 (23)	10 (7)	0.18	3.40 (2.13–5.43)	< 0.001
IN	64	51 (79.8)	11 (17)	2 (3.2)	0.12	1.99 (1.03–3.81)	0.037
PKD	41	32 (78)	7 (17.5)	2 (4.5)	0.13	2.32 (1.11–4.83)	0.023
Other	144	115 (79.5)	25 (17.5)	4 (3)	0.12	1.94 (1.16–3.24)	0.011
ESKD CVD-^c^	248	221 (89.1)	23 (9.3)	4 (1.6)	0.06	ref	-

Genotype distribution is shown as numbers (%)

*ESKD* End-stage kidney disease, *CVD* Cardiovascular disease, *CGN* Chronic glomerulonephritis, *DN* Diabetic nephropathy, *IN* Interstitial nephritis, *PKD* Polycystic kidney disease, *APOA5* Apolipoprotein A5, *MAF* Minor allele frequency

^a^OR was adjusted for age, sex, BMI, and hypertension

^b^Calculated versus ESKD CVD- group

^c^The same ESKD patient population with different primary kidney diseases, but without CVD

Table 5 shows the mean plasma TG levels according to the APOA5 rs662799 genotypes in CVD + and CVD- patients. As expected, the presence of C allele (CC and CT genotypes) was significantly associated with increased plasma triglyceride levels (*p* < 0.001 for both groups).

**Table 5:** Effect of the APOA5 rs662799 polymorphism on plasma triglyceride levels in ESKD patients with and without CVD

Group	Genotype	TG (mmol/l)	Difference vs. TT	95% CI	*p*
ESKD CVD +	TT (*n* = 423)	1.29 ± 1	ref		
	TC (*n* = 105)	1.82 ± 1.49	-0.530	-0.78 to -0.27	0.001
	CC (*n* = 24)	2.39 ± 1.36	-1.100	-1.56 to -0.63	> 0.001
	TC + CC (*n* = 129)	2.11 ± 1.42	-0.820	-1.05 to -0.58	> 0.001
ESKD CVD-	TT (*n* = 221)	1.07 ± 1.20	ref		
	TC (*n* = 23)	1.63 ± 1.46	-0.500	-1.08 to -0.03	0.038
	CC (*n* = 4)	2.19 ± 1.39	-1.120	-2.31 to 0.07	0.066
	TC + CC (*n* = 27)	1.91 ± 1.42	-0.840	-1.33 to 0.34	0.009

*ESKD* End-stage kidney disease, *CVD* Cardiovascular disease, *TG* Triglyceride

## Discussion

The incidence of CVD in patients with end-stage kidney disease is significantly increased. This can only in part be explained by conventional cardiovascular risk factors such as age, obesity, hypertension, hyperglycemia or hyperlipidemia [3]. The genetic background is an important element of CVD pathogenesis.

Plasma triglycerides are considered a risk factor for cardiovascular disease [6], thus the genes involved in TG metabolism and affecting serum triglyceride concentration are obvious candidates in CVD pathogenesis. Numerous previous studies reported associations between common variants of *APOA5* gene and differences in plasma TG levels, suggesting that they may be related to cardiovascular disease [25].

The -1131 T > C polymorphism is located in the promoter region of the *APOA5* gene and influences its expression level [8]. Our case–control study was performed to evaluate the potential association of the -1113 T > C (rs662799) polymorphism with a risk of developing CVD in end-stage kidney disease patients. We successfully genotyped 1300 individuals (800 ESKD patients and 500 healthy controls) with this polymorphism, finding the allele frequencies in the control group similar to those reported for other European populations [17, 26]. A distribution of the rs662799 polymorphism was compared between CVD + and CVD- ESKD patients. The results showed a strong association of the C allele and CC genotype with the occurrence of cardiovascular disease. The frequencies of the C allele and CC genotype were significantly higher in patients with CVD, with OR 2.41 and 3.13, respectively. This association of rs662799 polymorphism and increased risk of CVD is consistent with previous reports from different populations. Szalai et al. found a significantly higher frequency of the C allele in patients with coronary artery disease than in healthy individuals and suggested that this variant is an independent risk factor for CAD [17]. Hubacek et al. reported an association of the APOA5 polymorphism with a risk of myocardial infarction and underlined its role in determination of triglyceride levels [16]. In a Chinese study of 355 CAD patients, the rs662799 polymorphism was significantly correlated with an increased risk of CAD [20]. In another study, this polymorphism was associated with dyslipidemia and the severity of CHD in Chinese women [18]. The minor allele of the rs662799 was the risk factor for CAD in Chinese Han population [19]. The same polymorphism was also reported to be associated with susceptibility to ischemic stroke [27–29]. In contrast, some studies failed to identify the association of rs662799 polymorphism with cardiovascular disease [30–34]. The lack of association in some of these studies might be due to the fact that they included individuals with coronary atherosclerosis or minimal coronary stenosis as controls.

When the rs662799 polymorphism distribution was compared between our ESKD subgroups with the most frequent primary kidney diseases, the highest frequency of the C allele (18%) was observed in subgroup of patients with diabetic nephropathy, with OR (95% CI) 3.40 (2.13–5.43), *p* < 0.001. Similarly, an Iranian study revealed that the C allele of rs662799 was strongly associated with the risk of diabetic nephropathy in type 2 diabetes patients (OR 1.42, *p* < 0.01). The highest TG levels were observed in patients with CC + TC genotypes [35]. The association of this *APOA5* polymorphism with diabetic nephropathy was also reported in a study of Egyptian patients. The CC genotype carriers were more prone to a higher TG levels and development of DN. However, in this study only 20 DN + patients were involved and they were compared with healthy controls only and not with T2DM patients without nephropathy [36]. To the contrary, Baum et al. did not find any differences in rs662799 genotype distribution between 367 Chinese T2DM patients with DN and 382 patients without DN. They speculated that this result might be due to other pathways influencing the TG levels such as factors determining composition or clearance [37].

Apolipoprotein A5 plays an important role in triglyceride metabolism (synthesis and removal) and its gene expression level is associated with plasma TG concentration [38, 39]. Its effect on triglyceride metabolism is likely mediated by two mechanisms. One is an enhancement of the intravascular TG hydrolysis by activation of lipoprotein lipase. The other one is decreasing the serum concentration of TG by repressing the production of hepatic VLDL [8]. Both can be engaged in abnormal aggregation of lipids in the endothelial cells and formation of atherogenic plaques. Genetic polymorphisms can modify the effect of APOA5 on the lipid metabolism, causing the increased triglyceride levels.

In our study the minor allele of *APOA5* rs662799 was strongly associated with an increased risk of high plasma triglyceride levels. The presence of C allele (CC and CT genotypes) was significantly associated with increased plasma triglyceride levels (*p* < 0.001 for both CVD + and CVD- groups). This is consistent with previously published studies that reported higher triglyceride levels in carriers of rs662799 C allele [16, 17, 20, 32, 40]. According to Szalai et al. the C allele plays a role as modifier for plasma TG concentrations [17]. One of the plausible explanations for this effect is a stimulation of triglyceride hydrolysis mediated by lipoprotein lipase [Guardiola [39]. The effect of *APOA5* variants on plasma triglycerides is also dependent on other genetic (gene–gene interactions) and environmental factors [41, 42].

The strength of our study is very well determined definition of clinical phenotype of chronic kidney disease and comorbidities. However, as most of the case–control studies, the study has some potential limitations. Our findings showed a significant association of the *APOA5 rs662799* variant with CVD in ESKD patients, but they should be interpreted with caution. This being a retrospective case–control study, the data may be affected by a potential selection bias. The consecutive patients were recruited to limit this possibility. It is possible that some of the CVD- subjects, although asymptomatic, could have undetected subclinical atherosclerosis. Since the ESKD patient population presents with multiple coexisting diseases, some of these comorbidities might represent a confounding factor.

## Conclusion

The current study demonstrated the effect of the *APOA5* rs662799 polymorphism on plasma TG levels and its strong association with the risk of cardiovascular disease in ESKD patients. This novel finding, if replicated in a larger cohort of patients with ESKD, could play an important role in personalized medicine.

## Data Availability

The data presented in this study are not publicly available due to the conditions of acceptance. They are available on request from the corresponding author.

## Abbreviations

APOA5: Apolipoprotein A5
CHD: Coronary heart disease
CVD: Cardiovascular disease
eGFR: Estimated glomerular filtration rate
ESKD: End stage kidney disease
HDL: High density lipoprotein
HY: Hypertension
KDOQI: Kidney Disease Outcomes Quality Initiative
LDL: Low density lipoprotein
LDLR: Low density lipoprotein receptor
MAF: Minor allele frequency
PCR: Polymerase chain reaction
SNP: Single nucleotide polymorphism
SPSS: Statistical Package for Social Sciences
TG: Triglyceride
VLDL: Very low density lipoprotein
